# Diagnostic efficacy of FibroScan for liver inflammation in patients with chronic hepatitis B: a single-center study with 1185 liver biopsies as controls

**DOI:** 10.1186/s12876-022-02108-0

**Published:** 2022-01-29

**Authors:** Kaiping Jiang, Lei Zhang, Jianhong Li, Hongtao Hu, Qinghua Huang, Tengyu Qiu, Xiaoai Mo, Jian Ren, Wenqiang Guo, Yin Tao, Haijun Cui, Ying Zuo, Xuli Chen, Youqing Xie, Yanxing Li, Haimin Liang, Zhaohong Liu, Le Xie, Rongjun Mao, Qunfang Jiang, Kaizhou Huang

**Affiliations:** 1 Department of Hepatology, Foshan Hospital of Traditional Chinese Medicine Affiliated to Guangzhou University of Chinese Medicine, No.6 Qinren Road, Chancheng District, Foshan, 528000 Guangdong Province China; 2 Department of Ultrasound, Foshan Hospital of Traditional Chinese Medicine Affiliated to Guangzhou University of Chinese Medicine, No.6 Qinren Road, Chancheng District, Foshan, 528000 Guangdong Province China; 3 Department of Pathology, Foshan Hospital of Traditional Chinese Medicine Affiliated to Guangzhou University of Chinese Medicine, No.6 Qinren Road, Chancheng District, Foshan, 528000 Guangdong Province China

**Keywords:** Noninvasive diagnosis, Chronic hepatitis B (CHB), Liver inflammation, FibroScan, Liver stiffness measurement (LSM)

## Abstract

**Background:**

Noninvasive diagnostic technologies that can dynamically monitor changes in liver inflammation are highly important for the management of chronic hepatitis B (CHB) patients and thus warrant further exploration. This study assessed the diagnostic efficacy of FibroScan for liver inflammation in CHB patients.

**Methods:**

A total of 1185 patients were selected, and ultrasound-guided liver biopsy was performed within 1 month after the FibroScan test. The liver stiffness measurement (LSM), the reliability criteria (IQR/M) of LSM, the quality of liver biopsy (complete portal area, PA), and the liver inflammation grades were the main observation items of this study. With liver biopsy as the control, the diagnostic efficacy of FibroScan for liver inflammation in CHB patients was evaluated by receiver operating characteristic (ROC) curve analysis.

**Results:**

The grade of liver inflammation was positively correlated with the stage of fibrosis (*rho* = 0.829, *P* < 0.001). Different grades of inflammation will have significant rise in LSM values within the same fibrosis stage, and LSM values were positively correlated with liver inflammation grade and fibrosis stage, and the *rho* is 0.579 and 0.593 respectively (*P* < 0.001). Significant differences in the LSM of FibroScan were observed among different grades of liver inflammation (*P* < 0.0001). Liver biopsy (PA > 10) served as the control, and the cutoff point and the area under ROC curves (AUCs) of the LSMs for different inflammation grades were as follows: G2, 8.6 kPa, 0.775; G3 9.8 kPa, 0.818; and G4, 11.0 kPa; 0.832. With LSM cutoff values of 8.6 kPa, 9.8 kPa and 11.0 kPa, FibroScan showed certain diagnostic value for CHB patients with G2, G3 and G4 liver inflammation, especially those with G4 inflammation.

**Conclusions:**

The grade of liver inflammation was positively correlated with the stage of fibrosis, different grades of inflammation will have significant rise in LSM values within the same fibrosis stage**.** In addition to liver fibrosis, FibroScan could evaluate liver inflammation in CHB patients in a noninvasive manner.

**Supplementary Information:**

The online version contains supplementary material available at 10.1186/s12876-022-02108-0.

## Background

Chronic hepatitis B virus (HBV) infection, which causes nearly one million deaths each year, remains a major public health problem worldwide [1, 2]. The 69th World Health Assembly approved a Global Health Sector Strategy to eliminate viral hepatitis by 2030 after the World Health Organization (WHO) issued its first ever guidelines for the prevention, care and treatment of persons with chronic hepatitis B (CHB) infection. A modeling study estimated that the global prevalence of HBsAg was 3.9% in 2016 [2]. Among untreated patients with CHB virus infection, 15–40% progress to cirrhosis, which may lead to liver failure and liver cancer [3]. The prevention and treatment of CHB is so urgent that, in addition to drug research, researchers must explore rapid, dynamic and noninvasive diagnostic methods that could be used to monitor the occurrence and development of CHB. Noninvasive analyses of liver fibrosis might offer a promising strategy for earlier diagnosis [4], so noninvasive methods to evaluate liver fibrosis have been attempted. The most commonly used is transient elastography (TE), which estimates liver fibrosis by measuring liver stiffness [5]. Currently, FibroScan, which is based on TE techniques, is widely used across the globe and has become an important method for the assessment of liver fibrosis in patients with CHB [6–8]. The vast majority of patients with CHB will develop HBV-induced necrotic inflammation and progressive fibrotic liver processes [7], and patients with immune-active CHB display elevated alanine aminotransferase (ALT) activity and active hepatic necroinflammation [9], so the results of TE may be confounded by the severe inflammation associated with high ALT levels [10, 11]. The LSM value obtained with FibroScan was also found to correlate significantly with both liver fibrosis and necroinflammatory activity on biopsy, which was considered to explain the TE measurement of TE [12].

Some authors have stated that TE cutoffs should incorporate ALT levels, which fluctuate with inflammation in HBV infection [13]. In this case, why not evaluate the potential of FibroScan for the diagnosis of liver inflammation in CHB patients? Thus, based on the data of 1185 liver biopsy specimens, we conducted a single-center large sample study to assess the value of FibroScan for the diagnosis of liver inflammation in patients with CHB.

## Methods

### Study design and patients

The study protocol was approved by the Ethics Committee of Foshan Hospital of Traditional Chinese Medicine ([2016]006). All patients with CHB signed informed consent. All the data related to this study were registered on the International Clinical Trial Registry Platform (ChiCTR- DRD-16009773).

The study was carried out at Foshan Hospital of Traditional Chinese Medicine, Guangzhou University of Chinese Medicine, China (from May 2011 to May 2016). A total of 1185 patients with CHB were selected from the Department of Hepatology according to the clinical practice guidelines [7, 14]. Patients with any of the following were excluded: liver cirrhosis or liver cancer; high levels of total bilirubin (TBIL) (> 150 μmol/) or liver failure; complicated by metabolic diseases or autoimmune liver diseases; coinfected with HIV, HCV and HDV; abused alcohol or illegal drugs; a history of using nucleoside analogs, interferon, or other anti-hepatic fibrosis drugs within 24 weeks; receiving treatment with anti-inflammatory agents, hepatoprotectants or related drugs; mental diseases or other serious viscera diseases; overweight or central obesity patients (BMI ≥ 28.0 kg/m^2^); pregnant or lactating women.

ALT (normal range: 0–40 U/L) and TBIL (normal range: 0–17 μmol/L) were tested with an automatic biochemical analyzer, hepatitis B surface antigen (HBsAg) and hepatitis B e antigen (HBeAg) were detected by electrochemiluminescence immunoassays,and HBV DNA was analyzed via real-time PCR (detection limit: 2 log_10_ IU/mL).

### FibroScan

FibroScan® 502 (Echosens, Paris, France) test was performed on an empty stomach in the morning or more than 2 h after food intake in patients with CHB. FibroScan was performed independently by the 3 operators with medical background in our department. They had been trained by Echosens and obtained the training certificate.Each operator had more than 500 times of successful operation experience. The median value of 10 effective measurements was successfully tested 10 times [15]. The LSM results are expressed in kilopascals (kPa). In this study, the operators adhered to the following reliability criteria [16]: ratio of the interquartile range (IQR) to the median (M)(IQR/M) was less than 0.30, with less than 0.10 being regarded as the best, and a success rate no less than 60%, with over 90% being regarded as the best.

### Liver biopsy

Ultrasound-guided liver biopsy was performed within 1 month after the FibroScan test had been completed. A 16-gauge disposable needle was used for the liver biopsy so that the length of the extracted liver tissue was greater than 1.5 cm and included at least 6 complete portal areas (PAs). The obtained liver tissue samples were fixed with 10% neutral formaldehyde solution, embedded in paraffin, and sliced into 5 pieces continuously. Routine HE staining, Masson staining and reticular fiber staining were used for diagnosis. The liver fibrosis stage was determined according to the METAVIR system (S = fibrosis) [17]: S0 = no fibrosis, S1 = portal fibrosis without septa, S2 = portal fibrosis with rare septa, S3 = numerous septa without cirrhosis, and S4 = cirrhosis.

According to the Scheuer scoring system [18], liver inflammation in the patients with CHB was classified into five grades: G0, G1, G2, G3 and G4. Moreover, the degree of hepatic steatosis was divided into four grades [19]: 0 (< 5%), 1 (mild, 5–33%), 2 (moderate, 34–66%), and 3 (severe, > 66%).

The pathological diagnosis of all liver biopsy samples was completed by 2 pathologists in our hospital. If the independent pathological diagnosis results given by the two pathologists were consistent, a pathological report would be provided. If the results of the two pathologists were inconsistent, the director pathologist would give the final pathological diagnosis and provide a report after the two pathologists reviewed the pathological section of liver tissue and discussed it together.

### Statistical analysis

Statistical analysis was carried out by SPSS 20.0. Categorical variables are presented as absolute (n) and relative (%) frequencies, and continuous variables are presented as the means ± SD. The significance of each baseline difference was determined by the chi-square test, Fisher’s exact test, unpaired t-test, or Mann–Whitney’s test, as appropriate. A two-sided *P* value of less than 0.05 was considered to indicate statistical significance. The correlations were analyzed with Pearson’s correlation and the test of Spearman's rank-correlation coefficient.

Based on the gold standard for the pathological grade of liver biopsy tissue and carried out by MedCalc, the receiver operating characteristic (ROC) curve was plotted, and the area under ROC curve (AUC), cutoff-off point, sensitivity, specificity and false positive rate were calculated, respectively, to determine the efficiency of the LSM by FibroScan in diagnosing the degree of liver inflammation. The data were artificially divided into two parts. We considered G = 1 to be relatively healthy and G = 2, 3, and 4 to be diseased. The AUCs were all between 1.0 and 0.5. An AUC between 0.5 and 0.7 was regarded as low accuracy, an AUC between 0.7 and 0.9 was regarded as moderate accuracy, and an AUC was above 0.9 was regarded as high accuracy; an AUC equal to 0.5 indicated no diagnostic value.

## Results

### Patients

The main demographic and clinical characteristics of the 1185 patients with CHB included in the study are presented in Table 1. Among them, there were 894 (75%) male patients, 291 (25%) female patients, 658 cases of HBeAg-positive CHB and 527 cases of HBeAg-negative CHB. The median age of the HBeAg-negative group was 37 years, which was older than that of the HBeAg-positive group (31 years) (*P* < 0.001). The majority of patients were HBeAg positive or negative (*P* = 0.012). Among the 273 patients with hepatic steatosis confirmed by liver biopsy, not only was the incidence of hepatic steatosis in men (n = 230, 84%) higher than that in women (n = 43, 16%) (*P* < 0.001) but also the incidence of hepatic steatosis in the HBeAg-negative group (n = 142, 27%) was higher than that in the HBeAg-positive group (n = 131, 20%) (*P* = 0.004).

**Table 1:** Demographic and clinical characteristics of the CHB patients

		HBeAg		hepatic steatosis	
	Total (n = 1185)	Positive (n = 658)	Negative (n = 527)	n = 273	*P*
*Age*					
Median years	33 (15–67)	31 (15–60)	37 (17–67)	36 (16–67)	
Means ± SD	33 ± 9	31 ± 8^A^	37 ± 9^A^	36 ± 9	< .001^A^
*Sex*					
Female	291 (25%)	180 (27%)^B^	111 (21%) ^B^	43 (16%)^C^	.012^B^
Male	894 (75%)	478 (73%)^B^	416 (79%)^B^	230 (84%)^C^	< .001^C^
*ALT (U/L)*					
Means ± SD	153 ± 218	169 ± 216^D^	133 ± 220^D^		.005^D^
*TBIL* (μmol/L)					
< 17 μmol/L (n)	1010 (85%)				
17–50 μmol/L (n)	166 (14%)				
51–100 μmol/L (n)	6 (0.5%)				
101–150 μmol/L (n)	3 (0.25%)				
*HBV DNA* (log_10_ IU/L)					
Means ± SD	5.97 ± 1.96	6.77 ± 1.14^E^	4.97 ± 1.43^E^		< .001^E^
Hepatic steatosis	273 (23%)	131 (20%)^F^	142 (27%)^F^		.004^E^

Continuous variables are presented as the mean (SD) if normally distributed and median if not. Significant differences among them are reported as *P* values. ^A^^,^^B C^^,^^D^^,^^E^Significant differences (*P* ≤ .05) between groups are indicated as follows:

^A^For comparison between HBeAg-positive and HBeAg-negative patients

^B^For comparison of females and males who were HBeAg positive and HBeAg negative

^C^For comparison of CHB with hepatic steatosis between females and males

^D^For comparison of ALT between HBeAg-positive and HBeAg-negative patients

^F^For comparison of CHB with hepatic steatosis between HBeAg-positive and HBeAg-negative patients

The mean ± SD of ALT, which was higher (169 ± 216 U/L) in the HBeAg-positive patients than in the HBeAg-negative patients (133 ± 220 U/L) (*P* = 0.005), was 153 ± 218 U/L in all patients. The levels of TBIL in 1010 patients (85%) were lower than 17 μmol/L, those in 166 patients (14%) were between 17 and 50 μmol/L, those in 6 patients (0.5%) were between 51 and 100 μmol/L, and those in 3 patients (0.25%) were between 101 and 150 μmol/L. The level of HBV DNA (mean ± SD) in the HBeAg-positive patients was higher (5.97 ± 1.96 log_10_ IU/mL) than that (4.97 ± 1.43 log_10_ IU/mL) in the HBeAg-negative patients (*P* < 0.001).

### FibroScan

Based on a FibroScan test success rate of over 90%, the LSM values ranged from 2.4 to 72 kPa, with an average value of 11.96 kPa, and the LSM reliability results of IQR/M (%) were 70% (≤ 0.10), 23% (0.10–0.15), 4% (0.15–0.20), and 3% (0.20–0.30).

### Liver biopsy

Among the liver biopsy tissues of 1185 patients with CHB, 977 cases (82%) had more than 10 PAs, and 208 cases (18%) had fewer than 10 PAs; there was no statistically significant difference in terms of sex (*P* = 0.152). The inflammation grade and fibrosis stage of the liver tissues are shown in Table 2. From Table 2, we can see that the fibrosis stage and the inflammation grade are two-way ordered data, and the grade of liver inflammation was positively correlated with the stage of fibrosis (*the Spearman's rho* = 0.829,* P* < 0.001). Especially the liver inflammation of patients with cirrhosis (S4) is mostly G4, and G1 and G2 are rare. There was a statistically significant difference in the liver inflammation grade between the PA ≥ 10 group and PA < 10 group (*P* < 0.001) (Table 3).

**Table 2:** Inflammation grade and fibrosis stage in liver tissue of CHB patients

S, n = 1185	G, n = 1185			
	G1	G2	G3	G4
S0	42	13	1	0
S1	64	164	15	0
S2	10	220	148	0
S3	0	13	268	43
S4	0	1	43	140

*Spearman's* *rho* = 0.829, *P* < 0.001

**Table 3:** The liver inflammation grade among CHB patients with different PAs

Group	n	G, n				Mean rank
		1	2	3	4	
≥ 10 PA	977	60	321	421	175	634.31
< 10 PA	208	56	89	54	9	398.98

*Mann–Whitney U Z* = − 9.548, *P* = *.*000

There was a statistically significant difference between the PA ≥ 10 group and PA < 10 group in terms of liver inflammation grade (*P* < 0.001)

Hepatic steatosis was found in 1185 patients with CHB in 273 cases (23%), of which 205 cases (75%) were mild, 50 cases were moderate (18%), and 18 cases were severe (7%). There was no significant difference in the inflammation grade (*P* = 0.082) or fibrosis stage (*P* = 0.177) between the CHB patients with hepatic steatosis and those without hepatic steatosis.

### ALT

With 40 U/L as the baseline, the effect of ALT levels below 40 U/L and 2, 3, 5 and 10 times higher than the baseline on the LSM was observed. Differences in ALT levels did not affect the accuracy of LSM in the diagnosis of liver inflammation (*P* > 0.05).

### Changes of LSM values in different grades of inflammation within the same fibrosis stage

In CHB patients with liver fibrosis at stage S2 or above, the LSM values rised with the increase of the grades of liver inflammation. In S2 stage of liver fibrosis, there was significant difference in LSM values between different grades of liver inflammation (F = 10.664, *P* < 0.001). There was significant difference in LSM values between G1 inflammation and G3 inflammation (*P* = 0.01) and that between G2 inflammation and G3 inflammation (*P* < 0.001). In the S3 stage of liver fibrosis, there was significant difference in LSM values between different grades of liver inflammation (F = 6.194, *P* = 0.002). There was no statistical difference in LSM values between G2 inflammation and G3 inflammation (*P* = 0.051), there was significant difference in LSM values between G2 inflammation and G4 inflammation (*P* = 0.002) and that between G3 inflammation and G4 inflammation (*P* = 0.006). There was significant difference in LSM values between G3 inflammation and G4 inflammation in S4 stage of liver fibrosis (*P* < 0.001) (Table 4). Further analysis of the correlation between liver fibrosis stage, liver inflammation grade and LSM values in patients with chronic hepatitis B showed that LSM values were positively correlated with liver inflammation grade and fibrosis stage, the Spearman's Rho was 0.579 and 0.593, respectively (*P* < 0.001).

**Table 4:** Changes of LSM values in different grades of inflammation within the same fibrosis stage

S, n = 1185	G, n = 1185			
	G1 (LSM)	G2 (LSM)	G3 (LSM)	G4 (LSM)
S0 (56)	42 (7.31 ± 2.37)	13 (8.05 ± 2.28)	1 (5.6)	0
S1 (243)	64 (7.99 ± 2.54)	164 (8.22 ± 3.34)	15 (8.49 ± 2.77)	0
S2 (378)	10 (8.67 ± 1.44)	220 (8.85 ± 3.20)	148 (10.54 ± 3.97)	0
S3 (325)	0	13 (9.89 ± 2.66)	268 (13.97 ± 6.87)	44 (17.24 ± 10.24)
S4 (183)	0	1 (9.5)	43 (14.78 ± 6.39)	139 (20.87 ± 11.84)

S0 stage of fibrosis: t = − 1.002, *P* = 0.321

S1 stage of fibrosis: F = 0.211, *P* = 0.81

S2 stage of fibrosis: F = 10.664, *P* < 0.001; G1 versus G3, *P* = 0.01; G3 versus G2, *P* < 0.001

S3 stage of fibrosis: F = 6.194, *P* = 0.002; G2 versus G3, *P* = 0.051; G2 versus G4, *P* = 0.002; G3 versus G4, *P* = 0.006

S4 stage of fibrosis: G3 versus G4, t = − 4.358, *P* < 0.001

### The diagnostic efficacy of FibroScan (LSM) for liver inflammation in 1185 CHB patients

No G0 liver inflammation was observed in these CHB patients. The diagnostic efficacy of FibroScan (LSM, kPa) for liver inflammation was analyzed based on the sensitivity, specificity, false positive rate, cutoff points and AUCs for different inflammation grades (G1, G2, G3, G4).

- *G1-G2G3G4* There were significant differences in the LSMs between the liver inflammation grades when stratified by G1-G2G3G4 (*P* < 0.0001). The sensitivity was 56.82, the specificity was 83.62, the false positive rate was 16.38, the LSM cutoff value for group G2 was 9.6, and the AUC was 0.743.
- *G1G2-G3G4* There were significant differences in the LSMs between the liver inflammation grades when stratified by G1G2-G3G4 (*P* < 0.0001). The sensitivity was 74.36, the specificity was 74.71, the false positive rate was 20.69, the LSM cutoff value for group G3 was 9.7, and the AUC was 0.807.
- *G1G2G3-G4* There were significant differences in the LSMs (kPa) between the liver inflammation grades when stratified by G1G2G3-G4 (*P* < 0.0001). The sensitivity was 84.78, the specificity was 70.33, the false positive rate was 29.67, the LSM cutoff value for group G4 was 11.4, and the AUC was 0.838.

### The diagnostic efficacy of FibroScan (kPa) for liver inflammation based on different PAs in the liver tissue of CHB patients

**Fig. 1:**
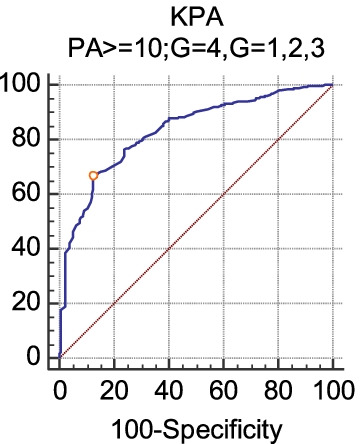
The diagnostic efficacy of FibroScan (kPa) for liver inflammation G4 (PA ≥ 10). When the inflammation grade was divided into two groups, the sensitivity, specificity, cutoff point and AUC of LSM (kPa) in diagnosing G4 were 87.43, 66.21, > 11 kPa and 0.832, respectively

**Fig. 2:**
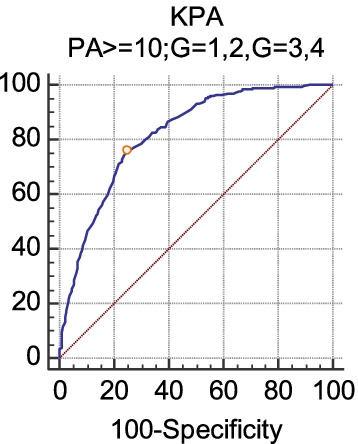
The diagnostic efficacy of FibroScan (kPa) for liver inflammation G3 (PA ≥ 10). When the inflammation grade was divided into two groups, the sensitivity, specificity, cutoff point and AUC of LSM in diagnosing G3 were 75.59, 75.51, ≤ 9.8 kPa and 0.818, respectively

**Fig. 3:**
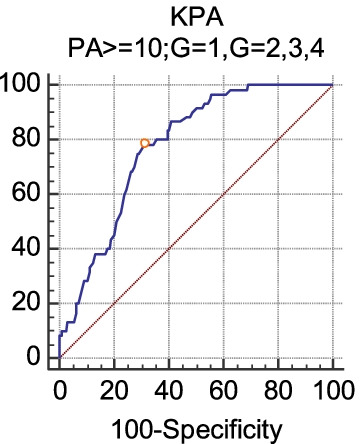
The diagnostic efficacy of FibroScan (kPa) for liver inflammation G2 (PA ≥ 10). When the inflammation grade was divided into two groups, the sensitivity, specificity, cutoff point and AUC of LSM in diagnosing G2 were 78.33, 68.70, ≤ 8.6 kPa and 0.775, respectively

PA ≥ 10: There were significant differences in the LSMs among the liver inflammation grades (*P* < 0.0001) (Table 5). The Youden index (0.536) and AUC (0.832) were largest when the inflammation grade was divided into two groups: G = 1, 2, 3 and G = 4. The sensitivity, specificity, cutoff point and AUC of LSM in diagnosing G4 were 87.43, 66.21, > 11 kPa and 0.832, respectively (Fig. 1). When the inflammation grade was redivided (G = 3, 4 and G = 1, 2), the sensitivity, specificity, cutoff point and AUC of LSM in diagnosing G3 were 75.59, 75.51, ≤ 9.8 kPa and 0.818, respectively (Fig. 2). When the inflammation grade was divided again (G = 2, 3, 4 and G = 1), the sensitivity, specificity, cutoff point and AUC of LSM in diagnosing G2 were 78.33, 68.70, ≤ 8.6 kPa and 0.775, respectively (Fig. 3).

**Table 5:** The diagnostic efficacy of FibroScan (LSM) for liver inflammation or fibrosis when PA ≥ 10

LSM	Group	Cutoff (positive)	Sensitivity (%)	Specificity (%)	Youden index	AUC	SE	Z	*P* (AUC = 0.5)
kPa ((PA ≥ 10)))	G = 4 G = 1,2,3	> 11	87.43	66.21	0.536	0.832	0.016	20.809	< 0.0001
	G = 1,2 G = 3,4	≤ 9.8	75.59	75.17	0.508	0.818	0.013	23.905	< 0.0001
	G = 1 G = 2,3,4	≤ 8.6	78.33	68.70	0.470	0.775	0.024	11.542	< 0.0001
	S = 0,1,2 S = 3,4	≤ 10.4	77.45	73.88	0.513	0.826	0.013	24.667	< 0.0001
	S = 0 S = 1,2,3,4	≤ 8.6	83.33	67.69	0.510	0.791	0.029	10.126	< 0.0001
	S = 4 S = 0,1,2,3	> 11	83.05	65.37	0.484	0.807	0.017	17.740	< 0.0001
	S = 0,1 S = 2,3,4	≤ 8.8	74.19	71.43	0.456	0.789	0.016	17.602	< 0.0001

PA of liver tissue ≥ 10: There were significant differences in the LSMs among the liver inflammation or fibrosis grades (*P* < 0.0001)

There were significant differences in LSM among the liver fibrosis grades (*P* < 0.0001) (Table 4). The Youden index (0.513) and AUC (0.826) were largest when the liver fibrosis staging was divided into S = 0, 1, 2 and S = 3, 4. The sensitivity, specificity, cutoff point and AUC of LSM in diagnosing S3 were 77.45, 73.88, ≤ 10.4 kPa and 0.826, respectively. When the liver fibrosis stage was divided into two groups, S = 1, 2, 3, 4 and S = 0, the sensitivity, specificity, cutoff point and AUC of LSM in diagnosing S1 were 83.33, 67.69, ≤ 8.6 kPa and 0.791, respectively. When the liver fibrosis stage was divided into S = 0, 1, 2, 3 and S = 4, the sensitivity, specificity, cutoff point and AUC of LSM in diagnosing S4 were 83.05, 65.37, > 11 kPa and 0.807, respectively. When the liver fibrosis stage was divided into S = 0, 1 and S = 2, 3, 4, the sensitivity, specificity, cutoff point and the AUC of LSM in diagnosing S2 was 74.19, 71.43, ≤ 8.6 kPa and 0.789, respectively.

## Discussion

Active inflammation promotes the development of fibrosis in CHB. Liver biopsy of chronic hepatitis showing variable necrotizing inflammation and/or fibrosis plays an important role in staging and grading CHB [20]. Despite its superiority of assessing both fibrosis and inflammation in CHB [8], liver biopsy is far from an ideal gold standard because of its invasiveness, risk of complications, patient discomfort and possible unavailability due to expertise requirements [21]. Staging CHB based on its severity using noninvasive tests such as elastography is important for guiding surveillance and assisting with treatment decisions [8]. Noninvasive tests are being increasingly incorporated into both national and international guidelines. With its good diagnostic accuracy for significant liver fibrosis and its excellent diagnostic accuracy for liver cirrhosis [22, 23], FibroScan has been widely applied. In addition to reflecting liver fibrosis, the LSM value by FibroScan (with liver biopsy as the reference standard) should also reflect changes in liver inflammation to some extent. Although it has been proven that necrotizing inflammation can lead to an increase in LSM in CHB [12, 24], more strong, persuasive clinical research evidence must be collected.

In our study, the main demographic and clinical characteristics of the 1185 patients with CHB were consistent with previous research reports, showing good representativeness [1, 7, 25]: the male patients accounted for 75%, the median age of HBeAg-negative CHB patients was older than that of HBeAg- positive CHB patients (*P* < 0.001), the incidence of CHB complicated with hepatic steatosis r in men was higher (84%) than that in women (16%) (*P* < 0.001), the incidence of the degree of hepatic steatosis in HBeAg negative group was higher than that in HBeAg positive group (*P* = 0.004), the means ± SD of ALT levels were higher in the HBeAg-positive patients than in the HBeAg-negative patients (*P* = 0.005), the patients with a normal level range of bilirubin accounted for 85% of the total while few patients had high bilirubin levels that affect liver stiffness, and the level of HBV DNA in the HBeAg-positive patients was higher than that in the HBeAg-negative patients (*P* < 0.001). More importantly, liver biopsy showed no difference in inflammation (*P* = 0.082) or fibrosis (*P* = 0.177) in patients with CHB, regardless of whether they were complicated by hepatic steatosis, which further supported the view that the presence of steatosis in CHB patients does not lead to differences in the histopathological findings [26].

Usually, the performance of noninvasive diagnostic methods for liver diseases is evaluated by calculating the AUC using liver biopsy as the reference standard [11].

Accordingly, the quality of liver biopsy specimens is very important. It is recommended that if applicable, the presence of fewer than 11 PAs be noted in the pathology report, with recognition that the diagnosis, grading, and staging may be incorrect due to an insufficient sample size [8, 27]. Good evidence shows that a biopsy containing 10 or fewer portal tracts results in underestimation of both the severity of the fibrosis stage and of the inflammatory grade in chronic viral hepatitis [28]. Therefore, for medical liver biopsies, the core of tissue should be intact and of sufficient size to demonstrate the lobular architecture of the liver over several portal tracts, which has been further emphasized in the recent guidelines of liver biopsy in clinical practice issued by the British Society of Gastroenterology, the Royal College of Radiologists and the Royal College of Pathology [29]. In our 1185 liver biopsy specimens of chronic hepatitis B, 82% had more than 10 PAs, and only 18% had fewer than 10 PAs, which provided a reliable guarantee for non-invasive diagnosis of liver inflammation or fibrosis by FibroScan.

A reliable TE assessment was defined as an assessment fulfilling three characteristics: a minimum of 10 readings, a success rate of measurements (“shots”) ≥ 60% and an IQR/median ratio (IQR/M) of ≤ 0.30 [16, 22, 30, 31]. The reliability of liver stiffness evaluations depend on the IQR/M according to the median liver stiffness level [17], so it is necessary to achieve a "very reliable" IQR/M (≤ 0.10) or the "reliable" IQR/M (0.10–0.30) in the FibroScan test to the greatest extent possible. With a test success rate of over 90%, the LSM reliability results of IQR/M in the 1185 patients with CHB were 70% (IQR/M ≤ 0.10), 23% (0.10 < IQR/M ≤ 0.15), 4% (0.15 < IQR/M ≤ 0.20) and 3% (0.20 < IQR/M ≤ 0.3), respectively. The grade of liver inflammation was positively correlated with the stage of fibrosis (the Spearman's rho = 0.829, *P* < 0.001). Especially the liver inflammation of patients with cirrhosis (S4) is mostly G4, G1 and G2 are rare.

In CHB patients with liver fibrosis at stage S2 or above, the LSM values rised with the increase of the grades of liver inflammation. In S2 stage of liver fibrosis, there was significant difference in LSM values between different grades of liver inflammation (F = 10.664, *P* < 0.001). There was significant difference in LSM values between G1 inflammation and G3 inflammation (*P* = 0.01) and that between G2 inflammation and G3 inflammation (*P* < 0.001). In the S3 stage of liver fibrosis, there was significant difference in LSM values between different grades of liver inflammation (F = 6.194, *P* = 0.002). There was no statistical difference in LSM values between G2 inflammation and G3 inflammation (*P* = 0.051), which may be related to a small number of patients with G2 inflammation; there was significant difference in LSM values between G2 inflammation and G4 inflammation (*P* = 0.002) and that between G3 inflammation and G4 inflammation (*P* = 0.006). There was significant difference in LSM values between G3 inflammation and G4 inflammation in S4 stage of liver fibrosis (*P* < 0.001). It seemed to indicate that different grades of inflammation will have significant rise in LSM values within the same fibrosis stage.In order to further confirm this, we also carried out Spearman rank correlation coefficient analysis. It showed that LSM values were positively correlated with liver inflammation grade and fibrosis stage, and the Spearman's rho is 0.579 and 0.593 respectively (*P* < 0.001). Therefore, LSM value is not only related to the stage of liver fibrosis, but also related to the grade of liver inflammation. That was to say, LSM value could also reflect the grade of liver inflammation to a certain extent.

Then, the sensitivity, specificity, misdiagnosis rate, cutoff point and AUC of LSM were compared individually, and significant differences in the LSMs were noted among different grades of liver inflammation in the 1185 CHB patients (*P* < 0.0001). The cutoff points and AUCs of LSMs for the diagnosis of G2, G3, and G4 were 9.6 kPa and 0.743, 9.7 kPa and 0.807, respectively, and 11.4 kPa and 0.838, respectively; that is, FibroScan could diagnose G2, G3, and G4 liver inflammation in CHB patients with LSM values of 9.6 kPa, 9.7 kPa and 11.4 kPa, respectively.

Considering that the number of PAs in liver biopsy tissues will affect the pathological diagnosis of inflammation or fibrosis of liver tissues, we also analyzed the diagnostic efficacy of FibroScan (LSM) for liver inflammation or fibrosis when PA ≥ 10 in the liver tissues of these patients. There were significant differences in the LSMs among different grades of liver inflammation (*P* < 0.0001). The cutoff points and the AUCs of the LSMs for the diagnosis of G2, G3, and G4 were 8.6 kPa and 0.775, 9.8 kPa and 0.818, and 11 kPa and 0.832, respectively. Significant differences were observed in the LSMs across the different stages of liver fibrosis (*P* < 0.0001). The cutoff points and the AUCs of the LSMs for the diagnosis of S2, S3, and S4 were 8.6 kPa and 0.789, 10.4 kPa and 0.826, and 11 kPa and 0.807, respectively; that is, FibroScan could diagnose G2, G3, and G4 liver inflammation in CHB patients with LSM values of 8.6 kPa, 9.8 kPa and 11.0 kPa, respectively. In addition, the efficacy of FibroScan for the noninvasive diagnosis of liver fibrosis, especially S4, was basically consistent with international reports or guideline recommendations [22, 32]. Most interestingly, the LSM cutoff point for G4 liver inflammation was 11.0 kPa, which was equal to that (11.0 kPa) for the diagnosis of S4 liver fibrosis. Therefore, we believe that FibroScan has certain potential for the noninvasive diagnosis of CHB, regardless of whether liver fibrosis or liver inflammation is being evaluated. Relevant studies had shown that LSM could diagnose different stages of liver fibrosis in patients with CHB after 78 weeks of antiviral treatment, and the decrease of LSM absolute value could reflect the remission of liver inflammation [33]. The latest study found that Liver inflammation activity over 2 (OR = 3.53) was an independent risk factor for misdiagnosis of fibrosis stage using FibroScan, patients with liver inflammation activity ≥ 2 showed higher LSM values using FibroScan and higher rates of misdiagnosis of fibrosis stage, whereas the diagnostic performance of FibroScan for different fibrosis stages was significantly lower than that in patients with inflammation activity < 2 (all *P* < 0.05) [34]. It is expert opinion that each patient becomes his or her own control, using the stiffness delta changes over time to evaluate the efficacy of the treatment or the progression of disease—remembering that the measurement reflects stiffness and not fibrosis [35]. Therefore, suppose of we see LSM 11 we consider that both liver inflammation and fibrosis exist. At this time, it is strongly recommended that these CHB patients should conduct liver biopsy to clearly distinguish the grade of liver inflammation and the stage of liver fibrosis, and establish the exact point of liver inflammation or fibrosis corresponding to LSM, so as to provide a real-time, dynamic and noninvasive reliable tracking means for long-term standardized treatment efficacy judgment or disease progress monitoring.

Treatment decisions for CHB sometimes depend on the presence of necroinflammation rather than fibrosis, so the challenge is now to decide on how best to apply validated noninvasive tests in CHB management [36]. ALT is used as a control liver test and serves as a nonspecific biomarker of liver injury, and serial testing of ALT levels is needed to guide treatment decisions for CHB patients [10]. Due to the discomfort of blood sample collection, the poor correlation with the degree of liver disease in CHB patients, and the fact that this measurement that may fail to identify patients with necroinflammatory activity or fibrosis [37, 38], serum ALT is still not the ideal biomarker for assessing the degree of liver injury in CHB patients.

Comparatively, owing to its noninvasive, rapid and dynamic nature, we should not overlook the superiority of FibroScan for the evaluation of liver inflammation in CHB patients. In some reports or guidelines on the noninvasive diagnosis of liver fibrosis by FibroScan, it has been suggested that the LSM cutoff value should be adapted to the ALT level since ALT levels tend to influence the LSM in CHB [39] and because ALT increases the LSM value in FibroScan and is an important factor or confounding factor affecting the accuracy of LSM, thus reducing its diagnostic efficiency [22, 40]. Since elevated ALT levels can reflect liver injury to some extent and necrotizing inflammation can lead to an increase in LSMs in CHB patients [24], why do we not deduce that the LSM value of FibroScan may reflect the degree of liver inflammation in addition to liver fibrosis? On the other hand, studies have shown that sustained HBV suppression with antiviral treatment can lead to a reduction in necroinflammatory activity and improvement in fibrosis stage, and CHB patients can have a significant reduction in liver stiffness during nuleos(t)ide analog treatment, even when there is little or no improvement in fibrosis according to the histologic findings [41, 42]. Therefore, the impact of ALT normalization by antiviral therapy has to be considered in the interpretation of the noninvasive liver fibrosis assessment results [11], which indicates that the LSM value of FibroScan reflects the recovery of liver inflammation rather than liver fibrosis in CHB patients after antiviral therapy at a certain period of time. Remarkably, different ALT levels did not affect the accuracy of the LSM for the diagnosis of liver inflammation in our study (*P* > 0.05), so the influence of ALT on LSM should not be considered too heavily, and more attention should be given to the effect of liver inflammation on LSM. Regardless of whether liver inflammation or fibrosis is present, a decrease in the LSMs of CHB patients are welcome.

In summary, a reliance on abnormal liver function tests unfortunately causes most patients with significant liver injury to be missed [4], so noninvasive diagnostic techniques are needed to aid in CHB diagnosis and treatment monitoring. As the earliest and most extensively evaluated elastographic method for liver stiffness, FibroScan has certain potential for the noninvasive diagnosis of liver inflammation in CHB. The liver inflammation of CHB is accompanied by the occurrence and development of liver fibrosis, which was also proved in this study. It is difficult for LSM to exclude liver inflammation as an important participant in noninvasive diagnosis of liver fibrosis. In that case, we could expand the new use of LSMs for noninvasive diagnosis of liver inflammation, which was the goal of this study. This study showed that FibroScan might be a noninvasive diagnostic method for liver inflammation in CHB patients, which was better not only to expand the application field of the noninvasive diagnostic techniques of Fibroscan, but also to analyze the clinical connotation of LSM from different levels. For example, a rapid decrease of LSM in a short time after antiviral therapy is not likely to represent the remission or reversal of liver fibrosis, but more likely to be the improvement of liver inflammation in our view.

## Limitations

There were still some defects in our study, especially how to adjust the impact of liver fibrosis on the readings were not clear, which is also the direction of further research in the future. On the other hand, this was a single-center retrospective study, so these findings need to be further verified by a multicenter prospective study.

## Conclusions

In conclusion, the grade of liver inflammation was positively correlated with the stage of fibrosis, different grades of inflammation will have significant rise in LSM values within the same fibrosis stage. Based on the good quality of liver biopsy specimens (PA ≥ 10), our single-center large sample data analysis showed that LSM cutoff points of 8.6 kPa, 9.8 kPa and 11.0 kPa were effective in the diagnosis of G2, G3 and G4 liver inflammation in patients with CHB, respectively. These results preliminarily showed that FibroScan could evaluate liver inflammation in CHB patients noninvasively, which is worthy of further clinical verification and improvement.

## Supplementary Information

**Additional file 1.** LSM corresponding to the liver inflammation grade, fibrosis stage and serum ALT and AST levels (examples for illustration).

## Data Availability

The datasets used and/or analyzed during the current study are available from the corresponding author on reasonable request.
