# Supplementary material for: Diagnostic efficacy of FibroScan for liver inflammation in patients with chronic hepatitis B: a single-center study with 1185 liver biopsies as controls
Source: BMC Gastroenterol. 2022 Jan 29;22:37. doi: 10.1186/s12876-022-02108-0 (PMC8800333; doi:10.1186/s12876-022-02108-0)
Supplement: Supplementary file 1 — Additional file 1. LSM corresponding to the liver inflammation grade, fibrosis stage and serum ALT and AST levels (examples for illustration). [file 12876_2022_2108_MOESM1_ESM.pdf]

The LSM values corresponding to the grade of liver inflammation (G), the stage of liver fibrosis (S) and the level of serum transaminase (ALT、AST) ---- Examples

Figure 1 LSM 11.6 kPa

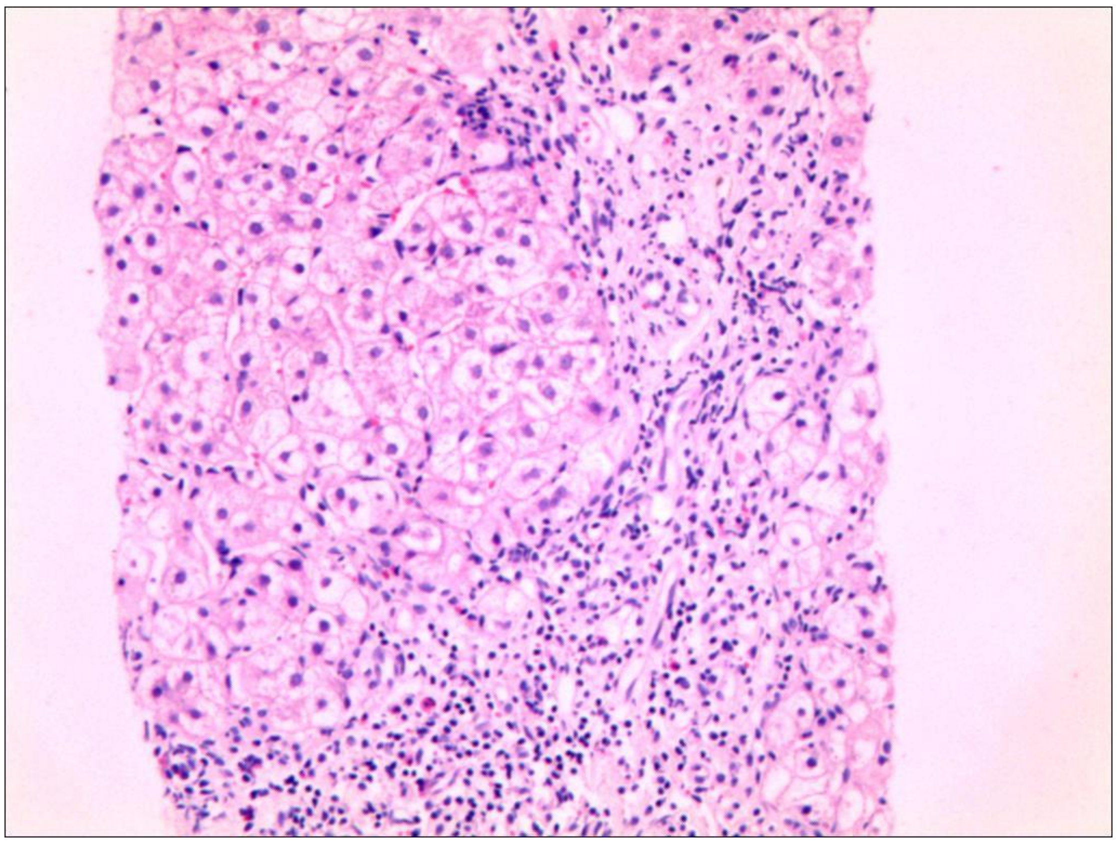

ALT 80.8 U/L
AST 55.2 U/L

G4

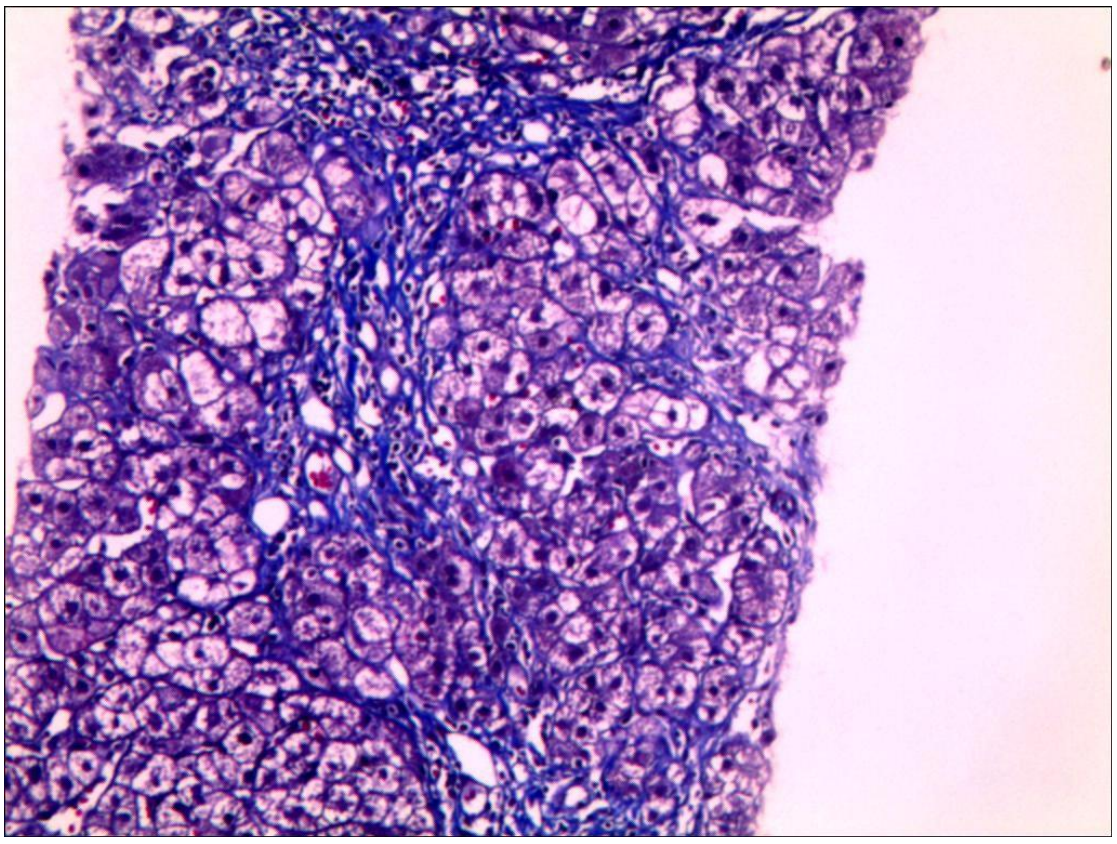

S3

Figure 2 LSM 12.7 kPa

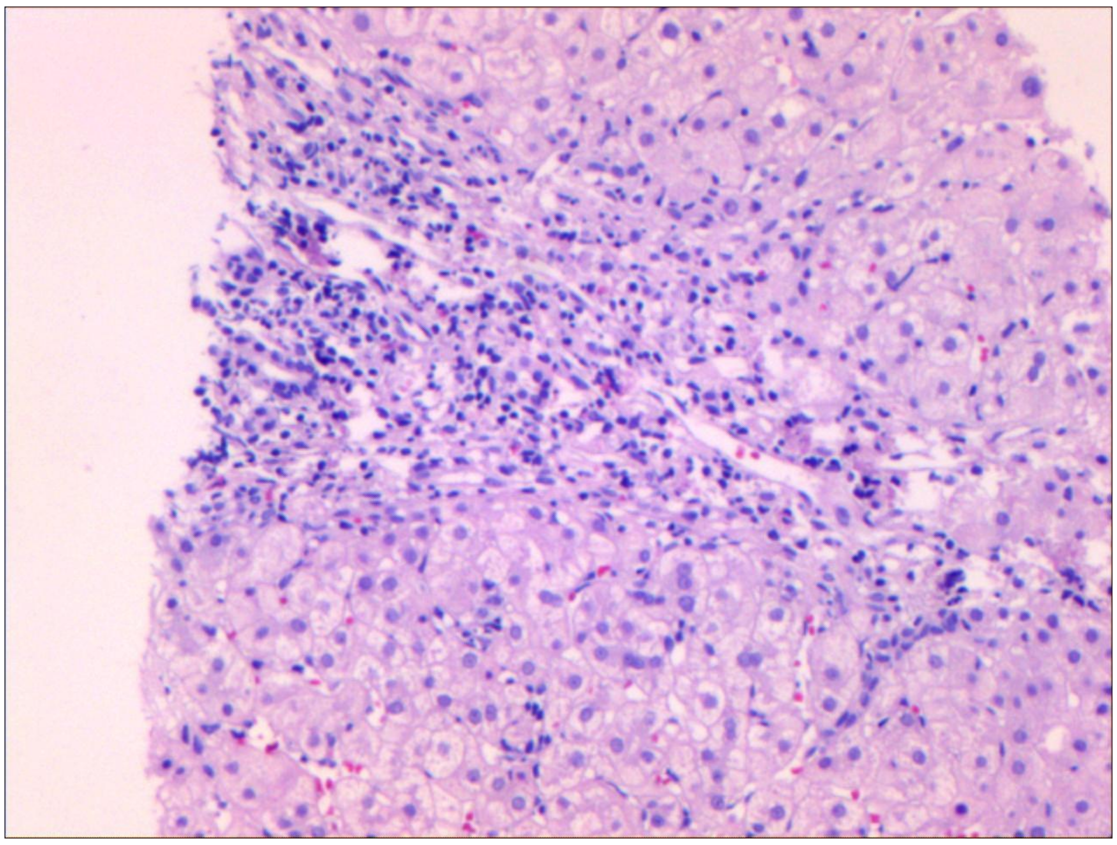

ALT 178.2 U/L
AST 90.4 U/L

G4

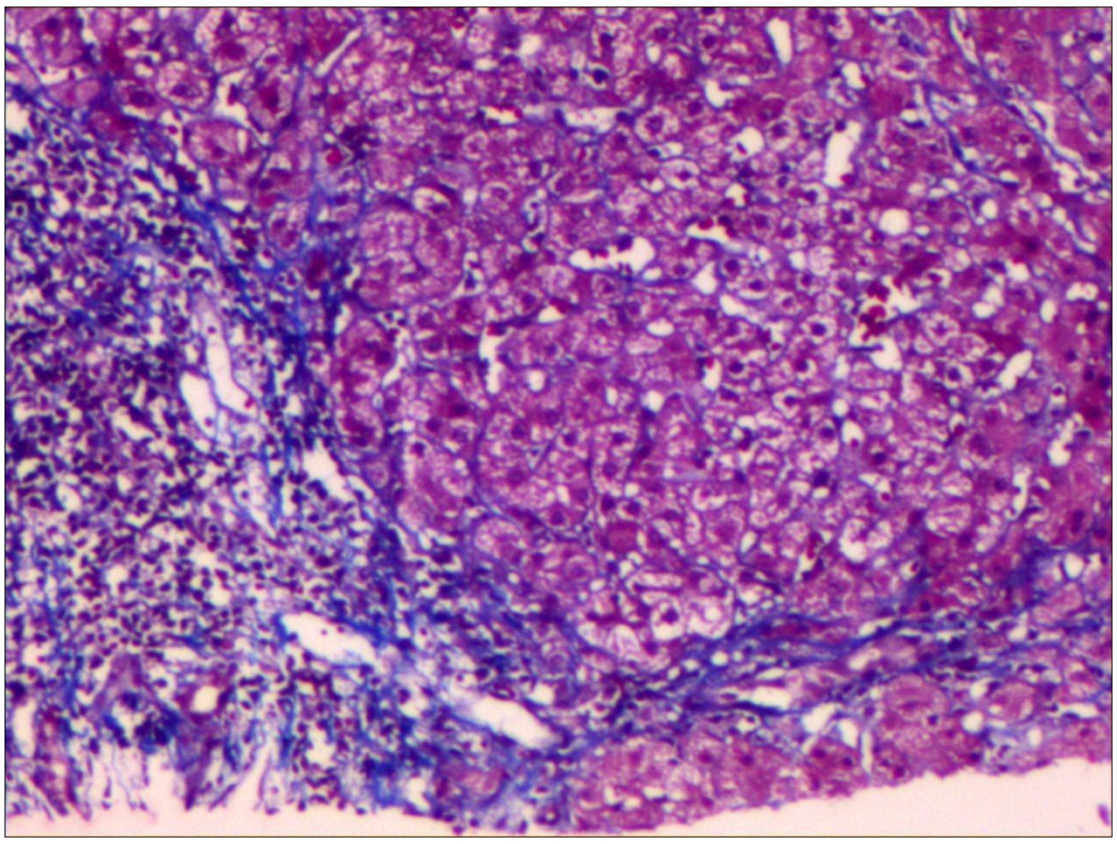

S3

Figure 3 LSM 16.3 kPa

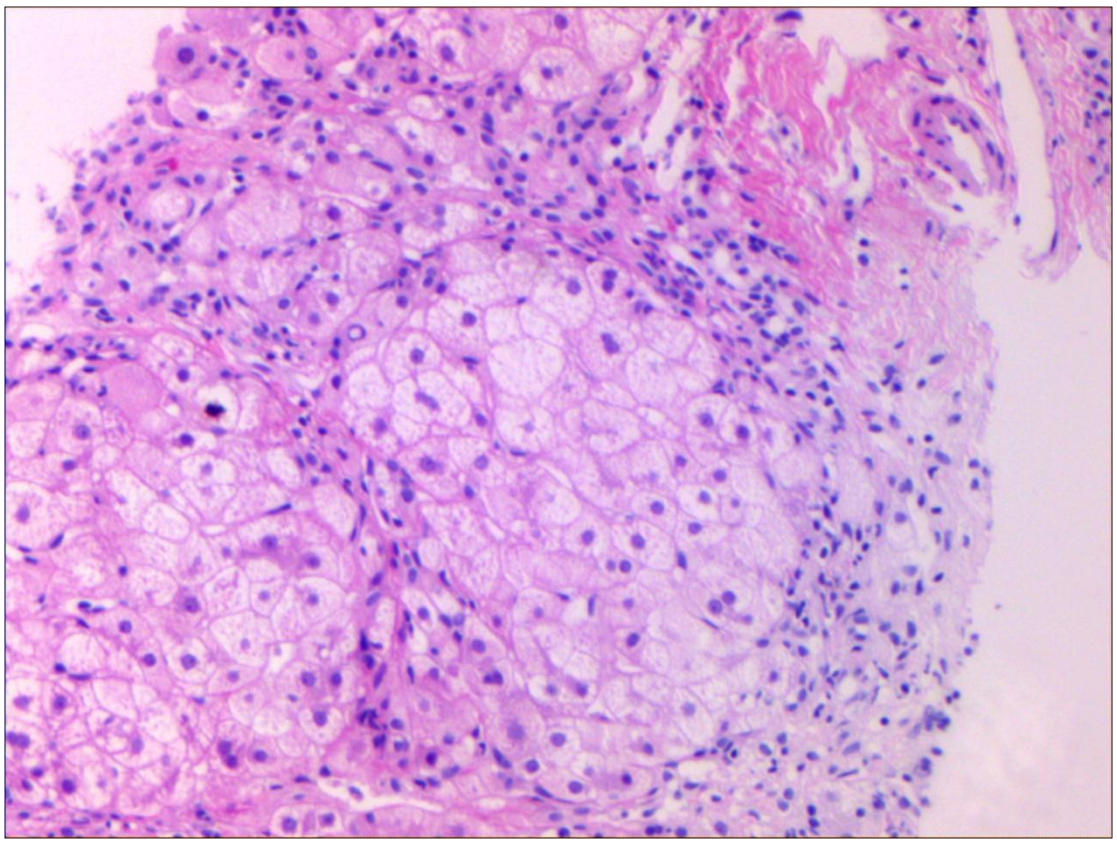

ALT 52.3 U/L
AST 36.9 U/L

G4

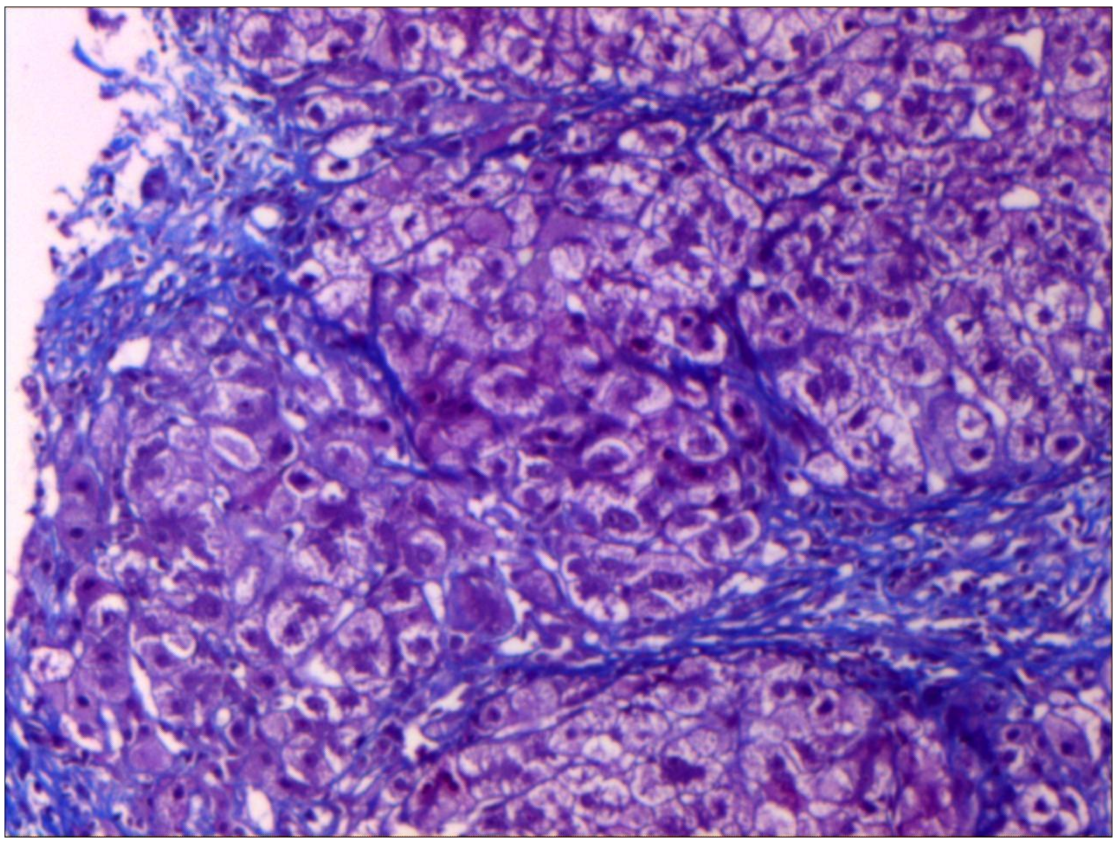

S3

Figure 4 LSM 17.3 kPa

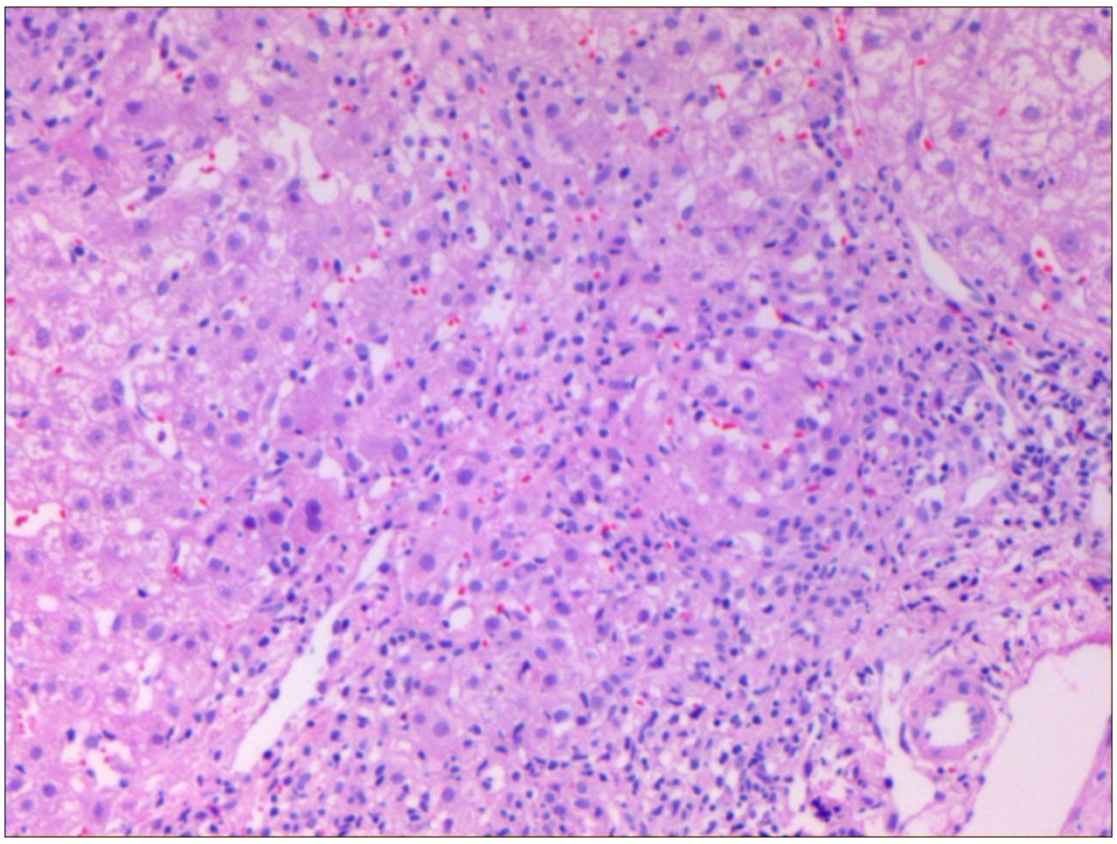

ALT 229.7 U/L
AST 148. 7 U/L

G4

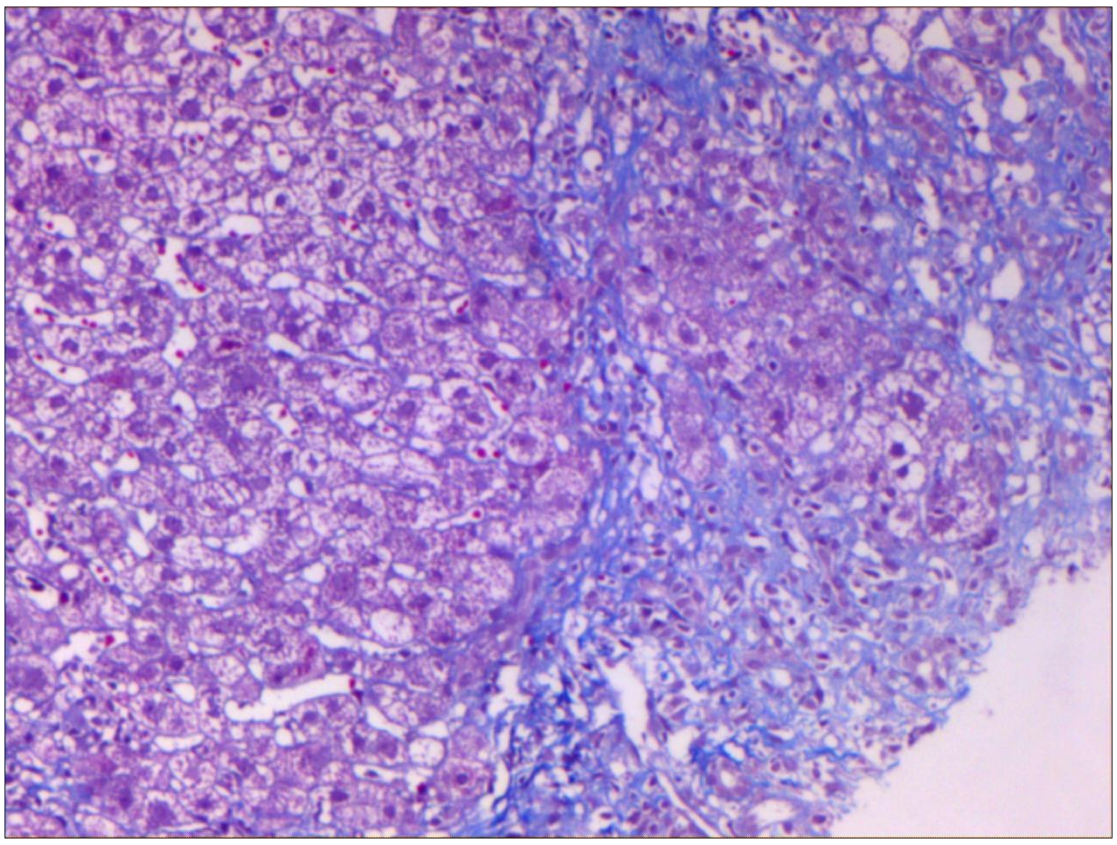

S3

Figure 5 LSM 17.6 kPa

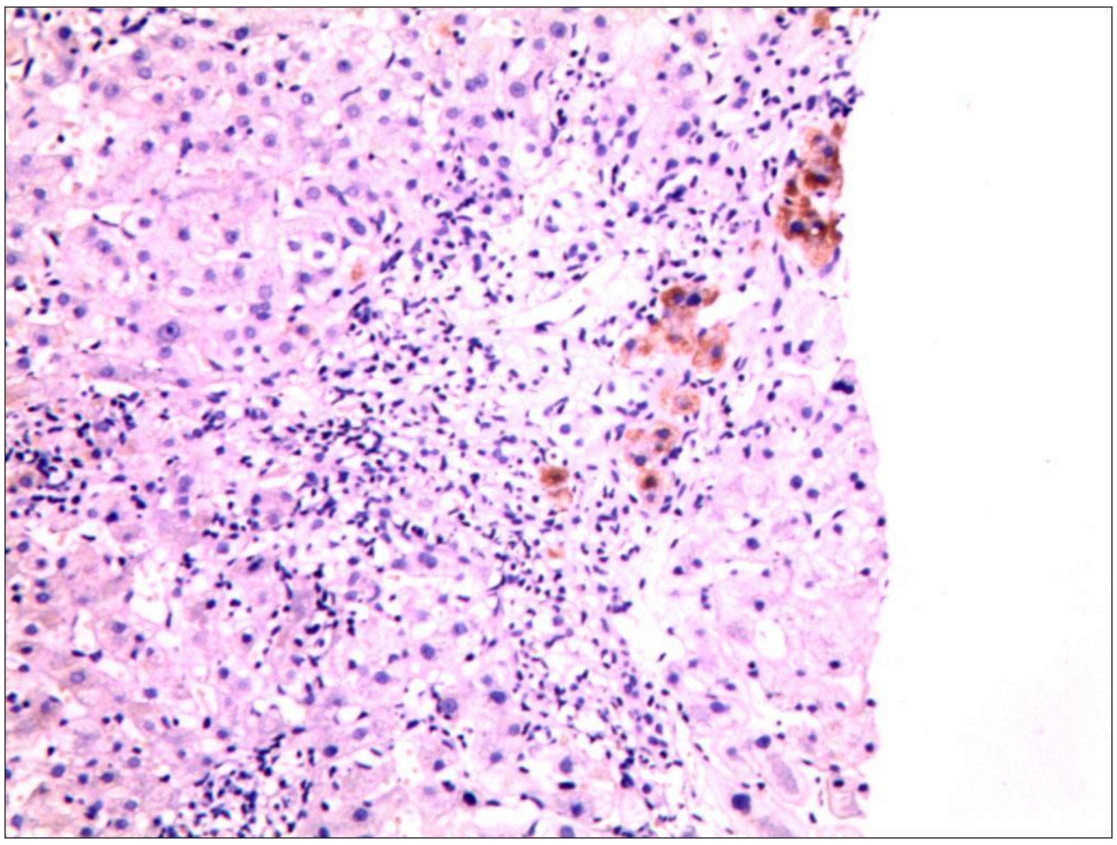

ALT 69.5 U/L
AST 60.4 U/L

G4

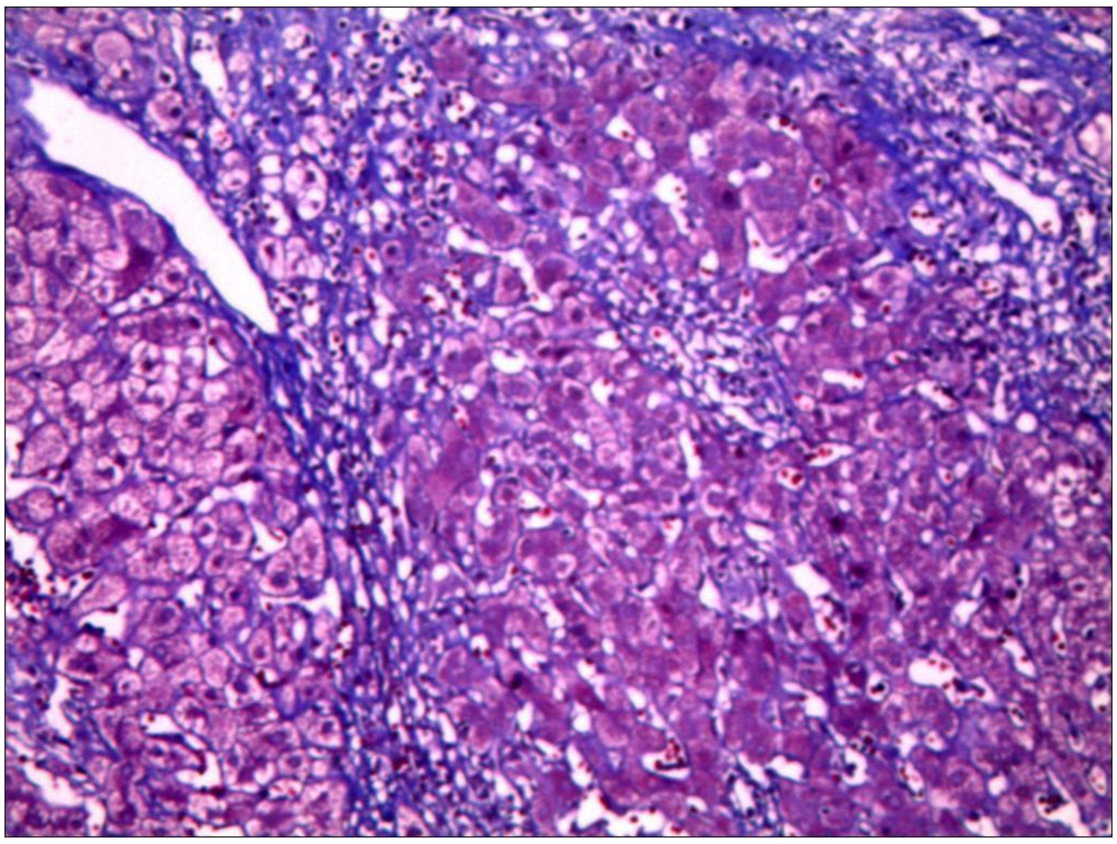

S3

Figure 6 LSM 12.6 kPa

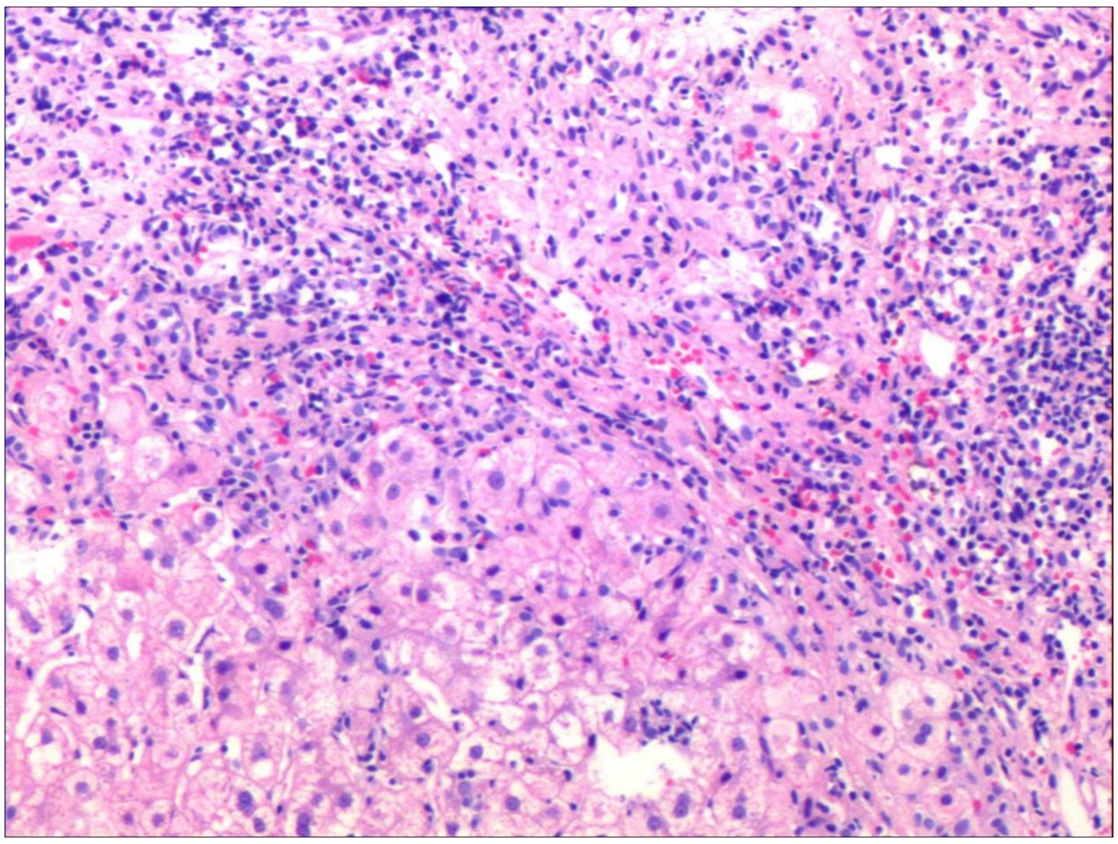

ALT 88.9 U/L
AST 53.8 U/L

G4

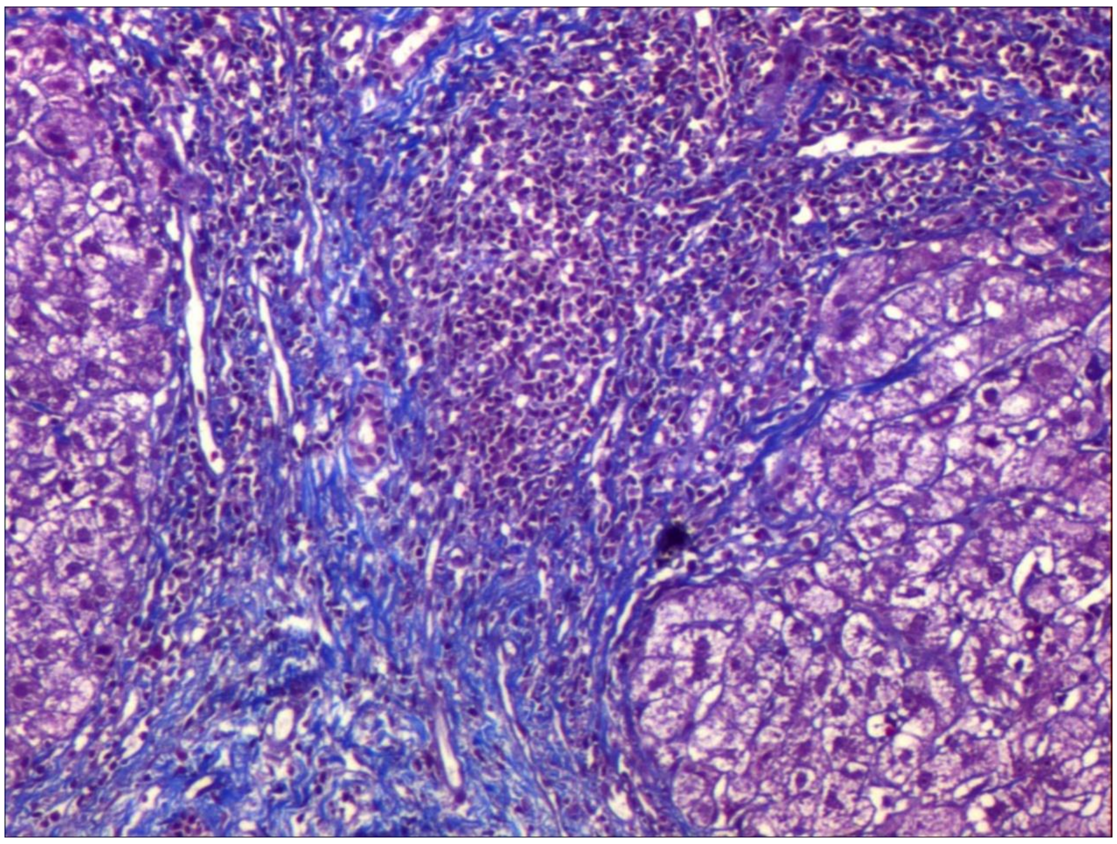

S4

Figure 7 LSM 16.6 kPa

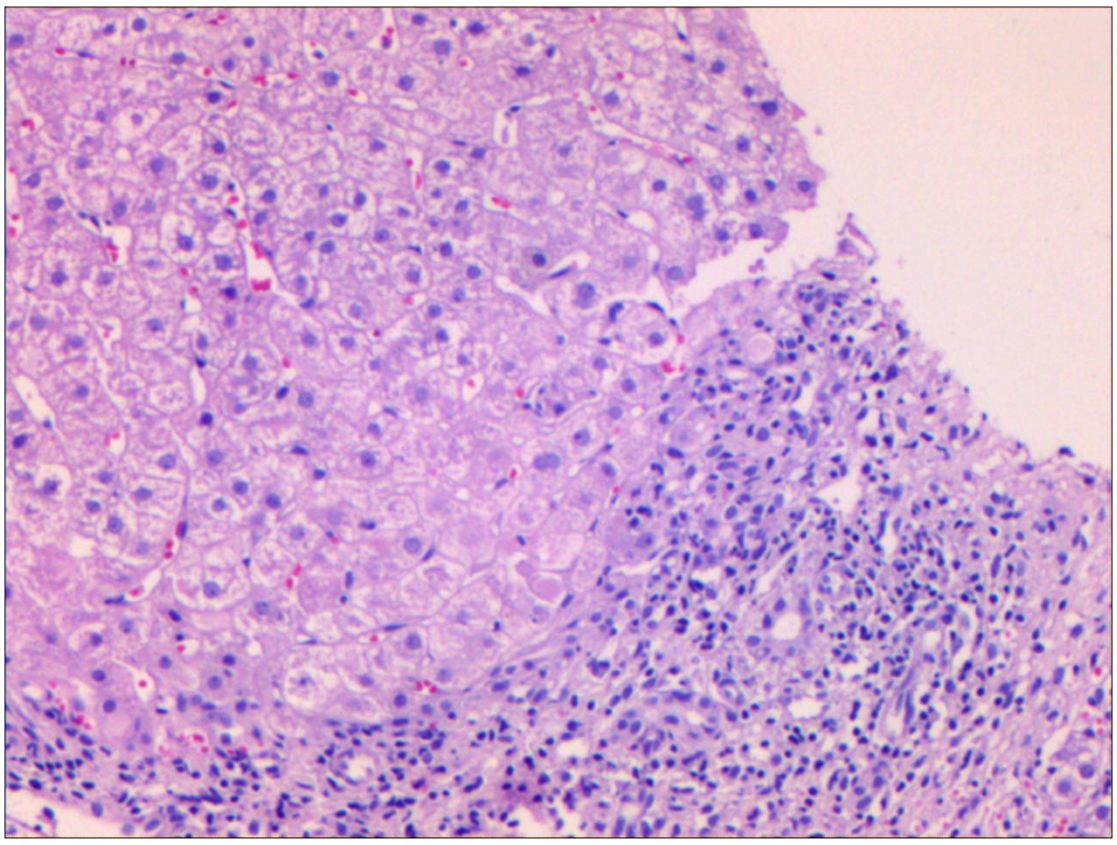

ALT 259.6 U/L
AST 279.9 U/L

G4

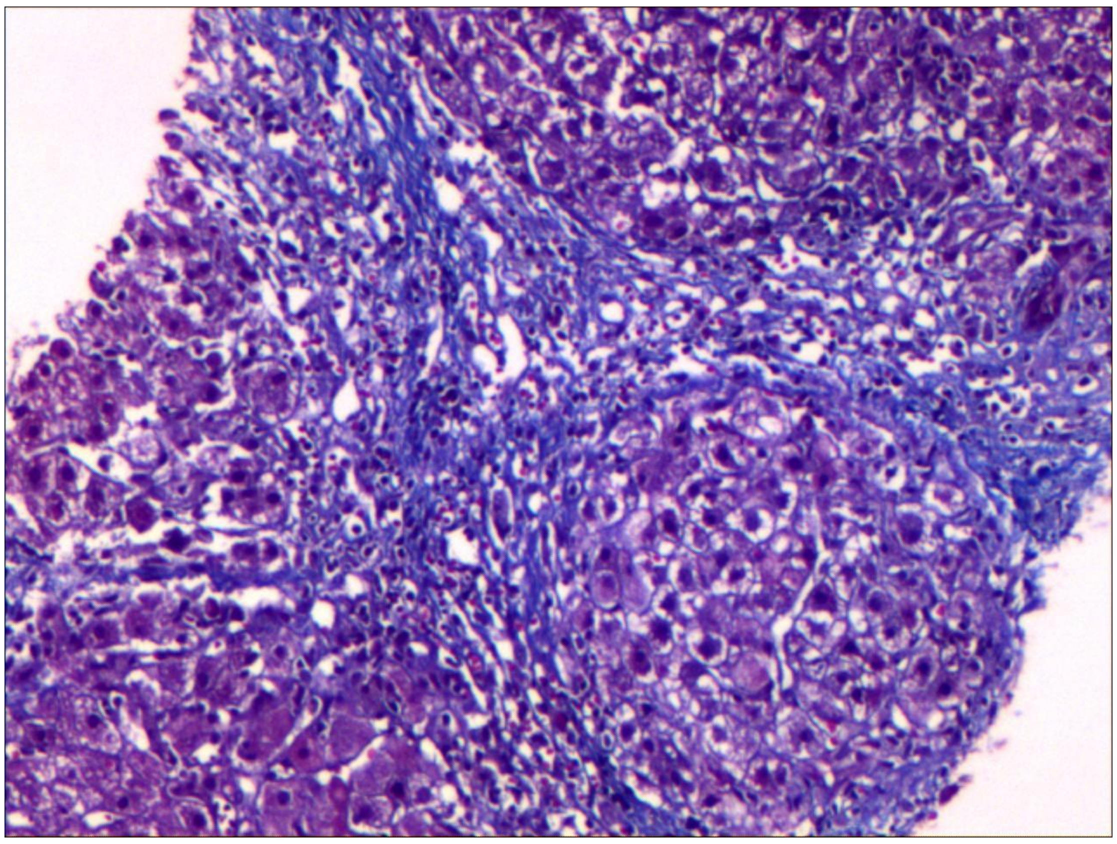

S4

Figure 8 LSM 20.2 kPa

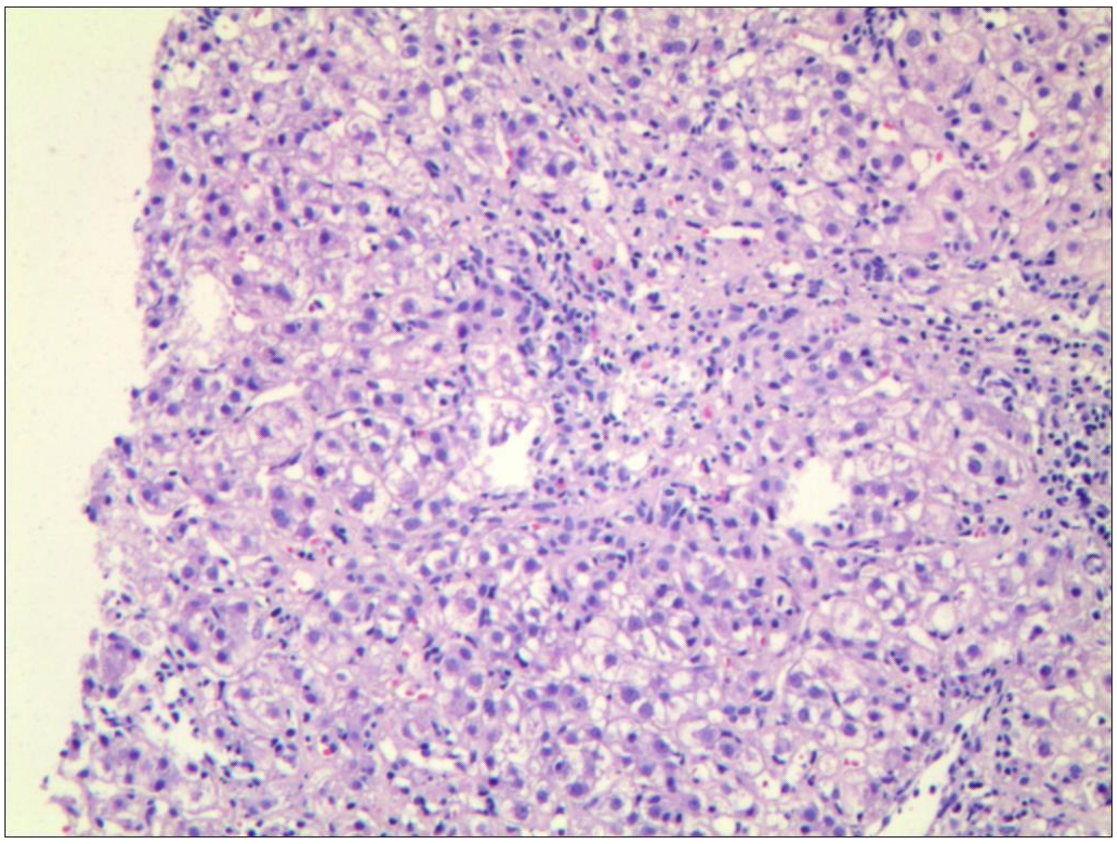

ALT 199.5 U/L
AST 125.2 U/L

G4

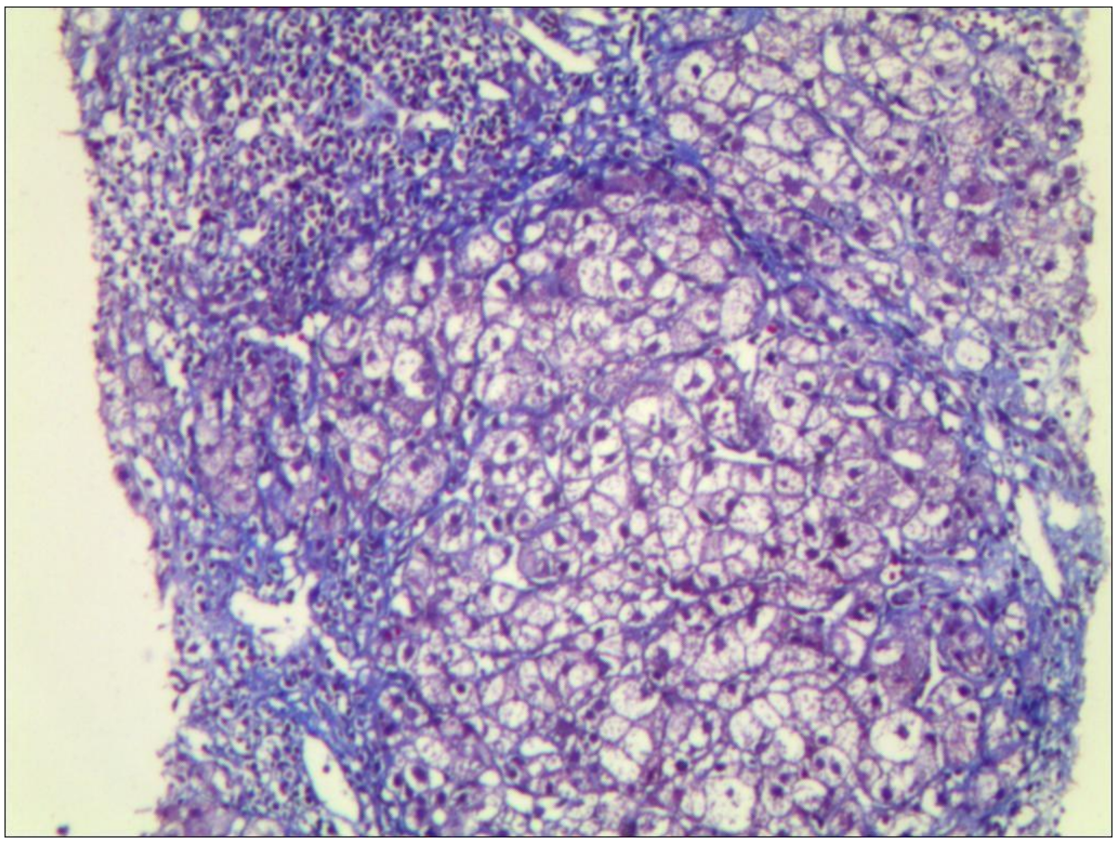

S4

Figure 9 LSM 21.1 kPa

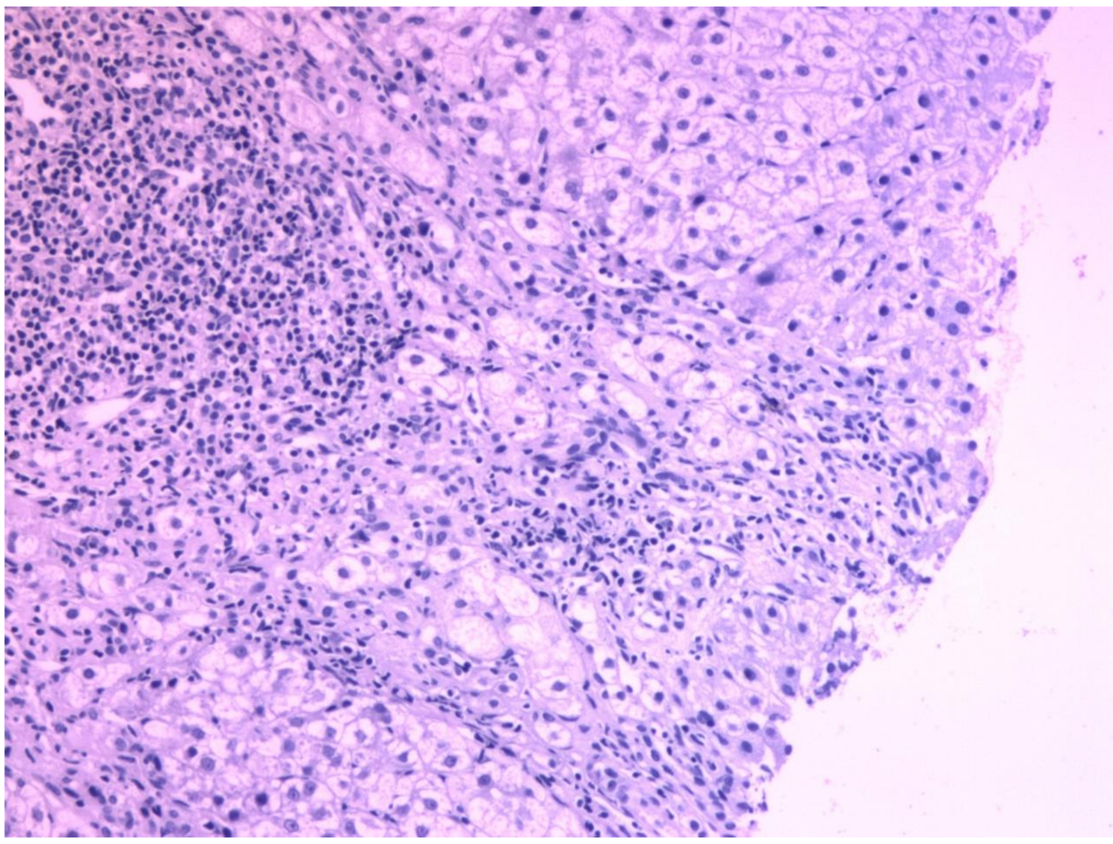

ALT 106 U/L
AST 54.1 U/L

G4

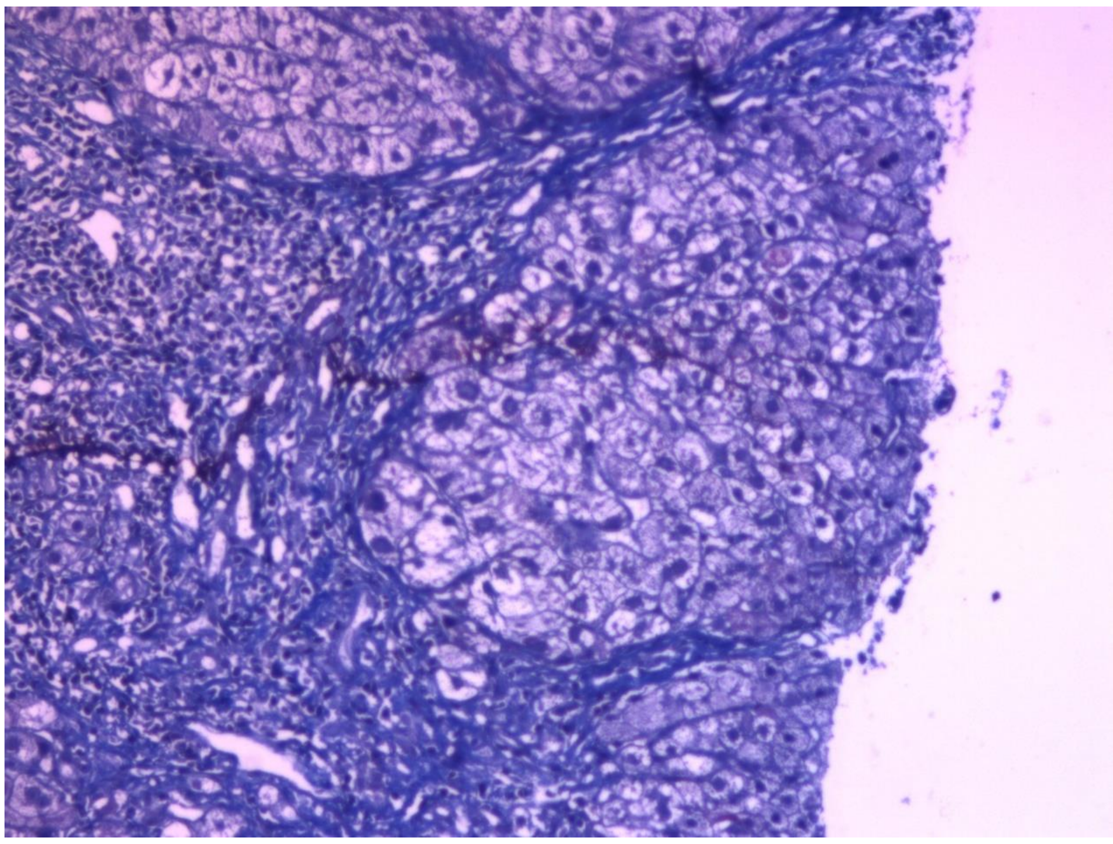

S4

Figure 10 LSM 27.0 kPa

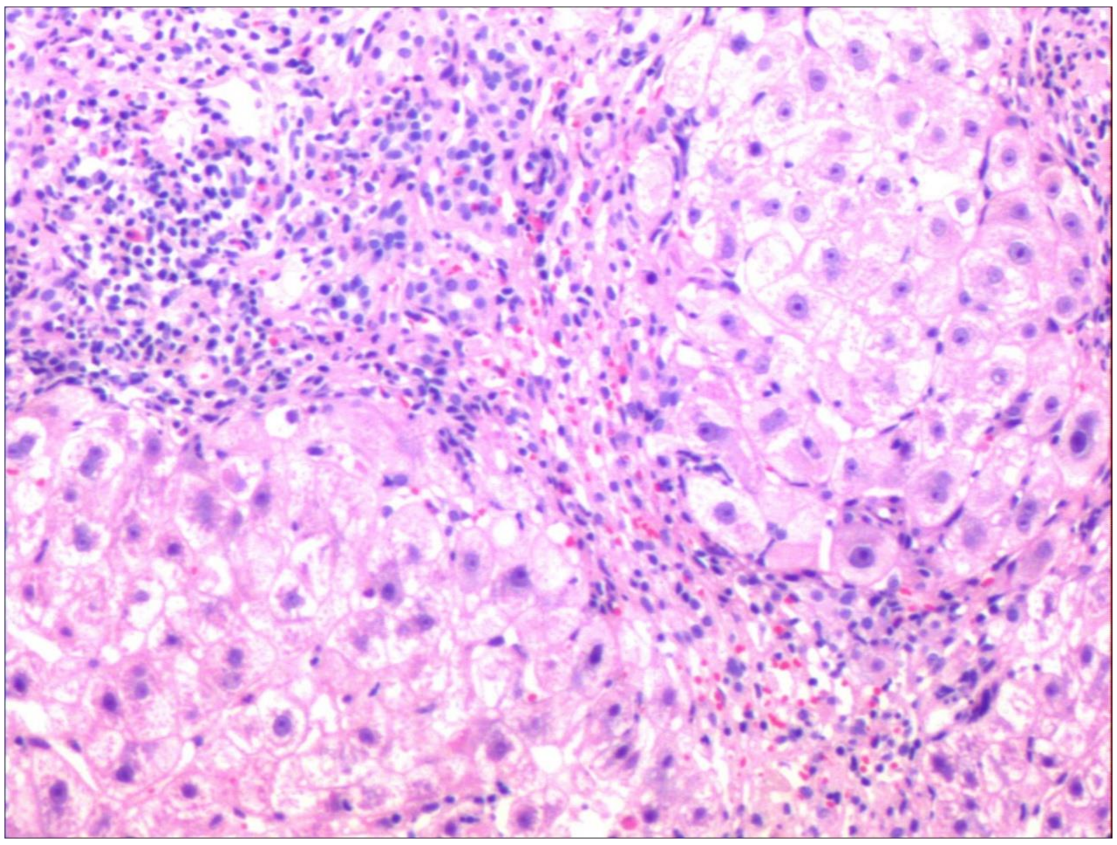

ALT 157.9 U/L
AST 85.5 U/L

G4

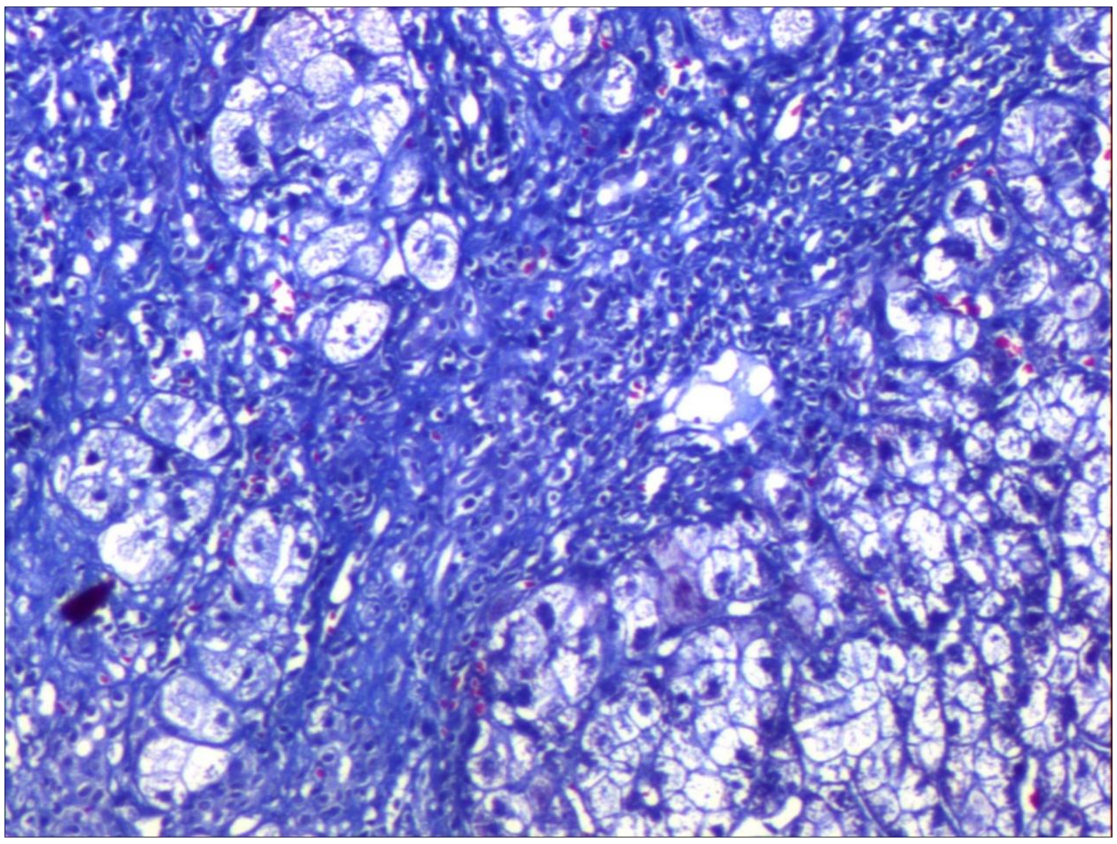

S4
